# Sir Andrew Clark—A Reminiscence

**Published:** 1894-06

**Authors:** 


### Sir Andrew Clark—A Reminiscence.

Miss Frances E. Willard writes the following interesting account of her visit to Sir Andrew Clark.

Although it is true that the article closes with a reference to a well-known preparation, yet the merits of the preparation itself warrant such a notice. Besides this the article is a most interesting one, in that it gives the best description we have seen of this great physician ; his methods ; and his directions for general treatment :

"This chief among the great physicians of London has just passed away in the sixty-seventh year of his age. He was Tennyson's physician and Gladstone's ; indeed, so great was his fame, that when, two weeks ago, he was stricken with paralysis, seven hundred messages of inquiry came to his family in a few hours. He was a small, slight man, of what we call the wiry type, and a remarkable illustration of what 'mind cure' can do for a person who is determined to live whether or no. It is said that forty years ago, when he sought admission as a physician in one of the London hospitals, the choice fell upon him in preference to a number of equally eager aspirants, on the basis that he was a 'delicate little fellow and would not live long anyway.' He was condemned to death in his youth by the verdict of physicians, but eluded the same by a novel process : he flung himself into the hardest kind of work, paying no attention to his fears and concentrated his forces altogether on his hopes.

"When I went to see him, he extended a hand white as a lady's and soft as velvet, and in a voice that matched the hand, went into a most careful diagnosis of my case ; beginning with heredity and ending with the last morsel I had tasted that morning. He followed me through every lane of life, ancestral and individual ; carefully examined my lungs and heart, saying (I think this was part of his mind-cure process), 'beautiful lungs, beautiful heart, no organic difficulty, over-work, nervous exhaustion. What you need is rest, pure air, cheerful companions, simple diet and no end of outdoors.'

"His manner was most reassuring and had in it a tender considerateness hardly to be expressed. When he asked to take the pulse or see the tongue, he prefaced the request with the words, 'My dear patient.' It was apparent that not only great skill and high character, but a most fortunate manner were the essentials of his success. He prescribed no medicine whatever, saying that he thought very little of it, and that old Mother Nature was the only true physician, and gave me some simple rules which seemed to me so good that I have had them copied for the benefit of any who may care to profit by the wisdom of a man both great and good, and a physician of unrivaled fame.

"At my request he wrote down three aphorisms he had used during our interview; 'Labor is the life of life;' 'Ease is the way to disease;' 'The highest life of an organ lies in the fullest discharge of its functions.' Here follow what he called his temporary general instructions;

"'On first waking in the morning sip about half a pint of water, cold or hot; on rising take a tepid sponge bath followed by a brisk general toweling. Clothe warmly and loosely. Avoid chilled, damp and passive exposure to cold. Take three simple, nourishing meals daily, and nothing between them. Breakfast at 8 to 9; plain or whole meal bread, or toast and butter with eggs, or fresh fish, or cold chicken, or game or tongue, fresh, not preserved, and toward the close of meal about half a pint of tea not infused over five minutes, or of cocoatina, or of coffee and milk.

"'Dinner from 1 to 2 o'clock; fresh, well-dressed meat, bread, potatoes, some well-boiled green vegetables, if it agrees, and either some simple farinacious pudding, or some simply cooked fruit. Toward the close of the meal drink water.

"'High tea five or six hours after dinner; whole-meal bread, or toast and butter, with broiled fish or cutlets, or a chop or cold meat, or cold chicken, and toward the close of meal about half a pint of black China tea, not infused over five minutes; cocoatina or cocoanibs may be substituted for tea if it is preferred, and if it agrees.

"'Nothing after this meal except that on going to bed you may sip a tumbler full of water, hot or cold.

"'Avoid soups, sauces, pickles, spices, salted, smoked, or otherwise preserved foods; pies, pastry, cheese, creams, ices, jams, dried fruits, nuts, raw vegetables, compotes, confectionery, malt liquors, cider, ginger beer, much liquid of any sort, and all sweet, sour and effervescent drinks.

"'Walk at least half an hour twice daily.

"'Retire as soon as possible after ten. See that your room is airy. Avoid self-notice and self-distrust. Shun ease and lead a full and regular, and an occupied life.

"'Whenever you have to speak at night be sure to lie down for an hour before tea.

"'Take nothing between meals.

"'Never take a sleeping draught.

"'Take as little medicine as possible; accept your sufferings; strength is perfected in weakness; in labor you will find life. If you are terribly run down some time, go away for a fortnight's rest, and with each meal take a teaspoonful of Fellows' Syrup of Hypophosphites.'"—*The National Medical Review.*

